# Estimating the Economic Effects of the Early Covid-19 Emergency Response in Cities Using Intracity Travel Intensity Data

**DOI:** 10.1007/s13753-022-00393-7

**Published:** 2022-02-18

**Authors:** Lijiao Yang, Caiyun Wei, Xinyu Jiang, Qian Ye, Hirokazu Tatano

**Affiliations:** 1 School of Management, Wuhan University of Technology, Wuhan, 430070 China; 2 Academy of Disaster Reduction and Emergency Management, Beijing Normal University, Beijing, 100875 China; 3 Disaster Prevention Research Institute, Kyoto University, Kyoto, 611-0011 Japan

**Keywords:** China, Covid-19, Emergency response, Economic impact assessment, Intracity travel intensity

## Abstract

In the early days of the Covid-19 pandemic, China implemented the most stringent and serious emergency response. To understand the effect of such an emergency response strategy on the economic system, this study proposed a simultaneous overall estimation method using intracity travel intensity data. The overall effect is represented by the difference between intracity travel intensity with and without the emergency response. Using historical data and time series analysis, we compared intracity travel intensity post China’s implementation of the emergency response with predicted intracity travel intensity without such a response. The loss rates, defined by the proportion of intracity travel intensity loss, were calculated for 360 cities within 33 provincial-level regions in China based on data availability. We found that 30 days after the emergency response, 21% of the cities saw over 80% recovery and 10% of the cities showed more than 90% recovery; 45 days after the emergency response, more than 83% of the 360 cities witnessed 80% recovery. The correlation between gross domestic production loss rate and travel intensity loss rate was studied quantitatively to demonstrate the representativeness of the intracity travel intensity loss rate. This indicator was also used to analyze the spatial and temporal patterns of the effects on the economy. The results of this study can help us understand the economic effects caused by the early Covid-19 emergency response and the method can be a reference for fast and real-time economic loss estimation to support emergency response decision making under pandemic conditions.

## Introduction

In December 2019, a novel coronavirus infection, now known as Covid-19, rapidly spread across the world, affecting many people grievously. In March 2020, the World Health Organization (WHO) declared the outbreak a pandemic, characterized by fast transmission speed, wide range of infection, and difficulty in controlling the disease. As the first affected city in China, Wuhan was decisively locked down on 23 January 2020. Buses, subways, ferries, and long-distance passenger transport were suspended, and the airport and all railway stations were shut down. Immediately, all provinces in China successively carried out the Level I emergency response (Tang et al. 2020; Tian, Li, et al. 2020). According to the nature, severity, and scope of impact, public health emergencies in China are classified into four levels (I, II, III, and IV), with severity decreasing from Level I to Level IV; accordingly, emergency responses are prepared for the four levels (Sun et al. 2018). The nationwide emergency response efficiently delayed the expansion of the virus by three to five days (Chinazzi et al. 2020), and significantly reduced the travel flow and concentration of people (De Vos 2020; Borkowski et al. 2021; Kim and Kwan 2021), especially during the Chinese Lunar Spring Festival, which witnesses the largest crowds every year (Xu et al. 2017).

However, the burden of stagnant economic activities and production behavior harmed the urban economy (Chakraborty and Maity 2020; Fernandes 2020; Nicola et al. 2020). From the demand side, the mass emergency response required people to consciously isolate themselves at home, which reduced consumption activities, such as shopping, dining, catering, travel, and entertainment (Qiu et al. 2020; Chang et al. 2021). From the supply side, road blocks interrupted the production and business activities of enterprises, thus causing serious disruptions in the supply chain as even factories were closed (Fernandes 2020; Pak et al. 2020; Veselovská 2020). The situation also affected risk perception around Covid-19 and both consumers and producers’ confidence in the economy was shaken (Lawal and Nwegbu 2020).

Qualitatively, an emergency response brings both benefits (preventing Covid-19 from spreading, and so on) and losses (affecting the socioeconomic systems, and so on). A quantitative analysis of benefit and loss is needed for policymakers. The overall effect of the emergency response should be estimated in a timely and reliable manner. The literature assessing the social and economic impacts of the Covid-19 pandemic, has generally discussed (1) the effectiveness of prevention of the spread of Covid-19 (Cartenì 2020; Han et al. 2020; Tian, Liu, et al. 2020); (2) gross domestic production (GDP) decrease after a statistical time scale (Maliszewska et al. 2020; Ozili 2020); (3) effects on the quarantined populations (Bodas and Peleg 2020; Guo et al. 2020); (4) impact on a specific industry (Rio-Chanona et al. 2020; Suau-Sanchez et al. 2020; Sigala 2020; Cui et al. 2021); (5) repercussions on global supply chains and the economy (Maliszewska et al. 2020); (6) efficacy of urban traffic restriction policies (Drew et al. 2020; Gao et al. 2020; Grantz et al. 2020; Zhou et al. 2020); and (7) experiences and challenges in early emergency response (Erkhembayar et al. 2020; Liu, Yue, et al. 2020). Given the broad impact of Covid-19, it is difficult to capture the overall consequences in a comprehensive and timely manner (Goode et al. 2021).

The basic principle of an emergency response to Covid-19 is limiting the movement of people. By assuming that any subsequent fall in consumption and production would be largely rooted in this restriction, a grounded measurement of the overall effect can be achieved by considering to what extent the emergency response limited people’s movement by using modern intracity travel intensity data. Specifically, the overall effect can be represented by the difference between intracity travel intensity with and without an emergency response. For this study, based on observed intracity travel intensity with emergency response during an early Covid-19 stage, and predicted intracity travel intensity without an emergency response through historical data and time series analysis, the overall loss rates for 360 cities within 33 provincial-level regions in China were calculated using available data. The spatial and temporal distribution of the effect of the emergency response during early Covid-19 was also analyzed. By comparing these data with the official GDP growth rate, the representativeness and statistical significance of intracity travel intensity loss rate to the economy is discussed. The method developed in this research can be a reference for quick loss estimation and emergency response decision making.

The remainder of the article is structured as follows: Sect. 2 introduces the data and methodology, Sect. 3 presents the results from the proposed method, Sect. 4 comprises the discussion, and Sect. 5 summarizes the conclusions.

## Data and Method

In this section, we introduce the data and the method this study adopted. We go through the various types of data and data sources in detail in the data section. Then, in the method part, we describe a method to estimate the economic impact of emergency response using intracity travel intensity data.

### Data

Population flow is a basic social activity, which can reflect the flow of material, capital, information, technology, and other advanced factors of production (De Haas 2010). Mobile phone data are usually used to capture population flow (Peak et al. 2018; Yabe et al. 2020). However, mobile phone data are generally expensive to access and involve privacy issues; researchers have encountered many obstacles in collecting large amounts of data for large-scale spatial population flow analysis (Bengtsson et al. 2015). Fortunately, with the emergence of various location-based service (LBS) providers, open big data based on sensitive information-filtered individual travel records are available (Xu et al. 2020). The LBS data are a kind of big data obtained through the global positioning system (GPS) and mobile phones, which have the characteristics of copiousness and vast areal coverage (Chao et al. 2018).

In China, Baidu is the largest electronic map and LBS provider, and thousands of mobile applications avail themselves of its services (Ming et al. 2020). Baidu Map has more than 500 million active users and receives on average billions LBS request logs every day (Huang et al. 2022). Compared with other open big data, Baidu migration big data based on LBS not only provide a relatively uniform data type but also conduct a timely, dynamic, complete, and accurate monitoring of national population travel and urban vitality; thus, the results of the data analysis are more reliable (Bao et al. 2021). Baidu migration big data have been used in many research fields, such as in exploring the spatiotemporal patterns of population mobility and related factors (Li et al. 2016; Wei and Wang 2020), studying the effects of human travel restrictions on air quality during the 2020 pandemic (Ming et al. 2020; Zhu et al. 2020), and predicting people’s daily travel demands to assist traffic decision making (Wang et al. 2019; Zhang, Xiao, et al. 2020).

For this study, we gathered intracity travel intensity data specifically from Baidu Map Smart Eye—Baidu Migration Big Data.^1^ The intracity travel intensity is the ratio of the number of people traveling in the city to the number of people living in it (Yuan et al. 2020). Taking the city as the basic statistical unit and one day as the time interval, we collected the intracity travel intensity data of 360 Chinese cities. These cities include all of China’s provincial-level municipalities—the 4 municipalities directly under the central government, 2 special administrative regions, 30 autonomous prefectures, 7 regions, 292 prefecture-level cities, 3 leagues, and 22 prefectures directly affiliated with the provinces. Owing to the lack of data, Taiwan was not included in the study. Since we focused on the first quarter of 2020, when a nationwide emergency response was first implemented, the sample data were collected over two time periods: January–April 2019 and January–April 2020.

In addition, we collected spatial GDP data, road data, the administrative map, the number of Covid-19 infection cases, and the length of the first-level emergency response. The GDP growth data for the 33 provincial-level regions in the first quarter of 2020 and GDP data for all prefecture-level cities in China in 2019 were obtained from the official websites of the provincial and municipal statistics bureaus. As GDP growth data in the first quarter of 2020 are all negative, we defined GDP growth in this study as the GDP decrease rate or loss rate. The road data and administrative map are available for download from the National Catalogue Service for Geographic Information website.^2^ Data on the number of Covid-19 infection cases were obtained from the national and provincial health committees, while the length of the first-level emergency response was gathered from provincial government websites.

### Method

The following subsections present our research methods, including the conceptual map of the economic effect of the emergency response, the method of predicting intracity travel intensity without emergency response, and the method of estimating the loss rate of intracity travel intensity.

#### Conceptualization

**Fig. 1:**
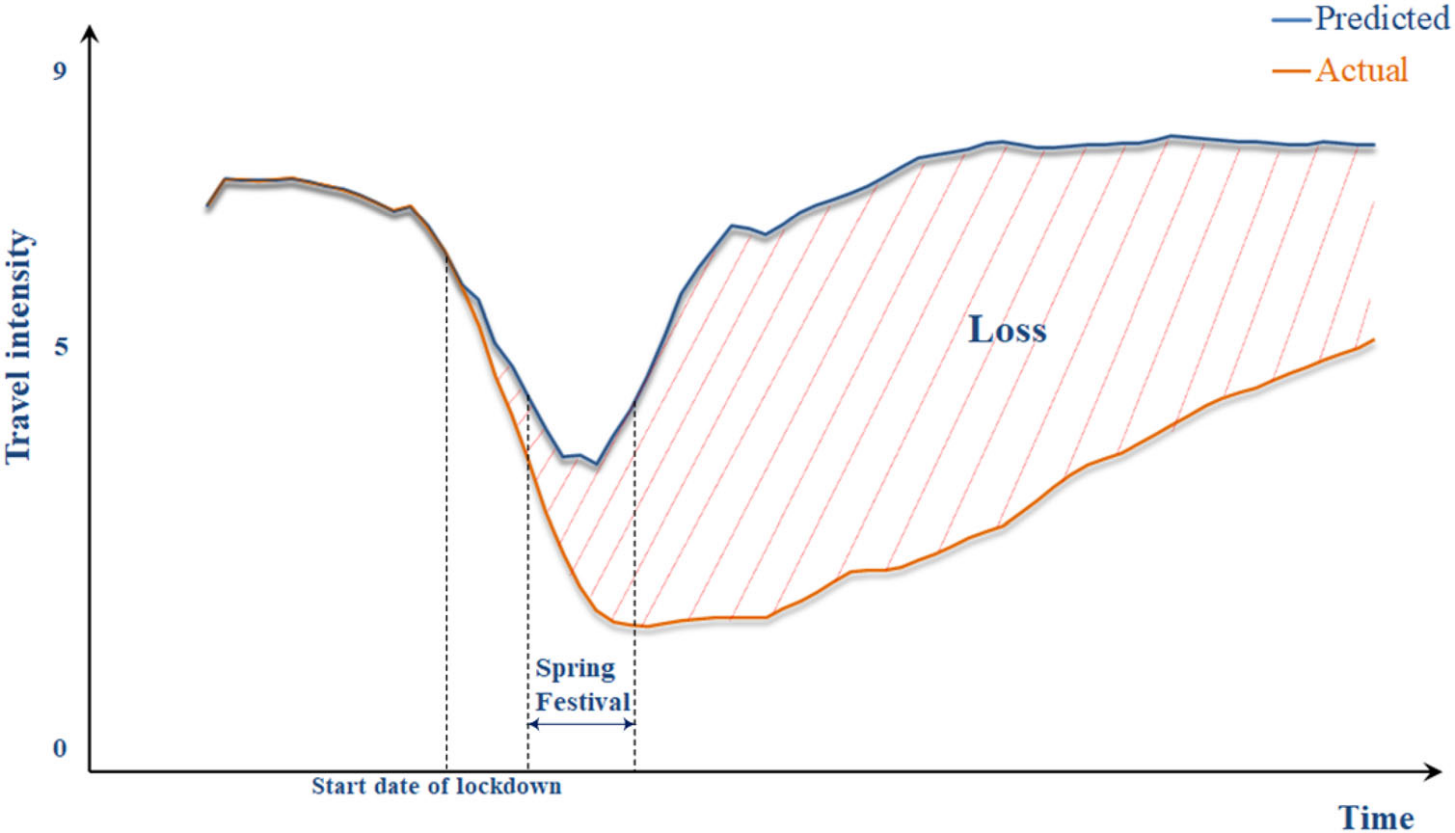
Conceptual map of the effect of the Covid-19 emergency response measured by intracity travel intensity

Our conceptual map is based on the basic structure from Yang et al.’s (2016a) natural hazard-related disaster impact assessment (Fig. 1).

The vertical coordinate is the index of intracity travel intensity. The horizontal coordinate is the time dimension. The solid lines represent the trend of intracity travel intensity. If no emergency response occurs, the trend of the intracity travel intensity will be a solid blue line representing the “normal” state. This line becomes a solid red line when a Covid-19 emergency response occurs, representing a period of drop, stagnation, or recovery. The shaded area between the solid blue and red lines is the difference between the intracity travel intensity with and without emergency response. Here, we used it to represent the overall economic effect of the emergency response.

#### Prediction of Intracity Travel Intensity Without Emergency Response

The trend of intracity travel intensity with emergency response in 2020 is based on real observations. We derive the “without” emergency response trend in the following way. The intracity travel intensity data are time series data with a seasonality of seven days. The seasonality does not change in a statistically significant way with time. We consider that the annual trends between two periods should not be significantly different, provable by a Wilcoxon test. We employed an additive decomposition method to analyze the data (Kendall and Stuart 1983). Based on the 2019 intracity travel intensity, we estimated the 2020 intracity travel intensity without an emergency response as follows.

*Step 1 Decomposition of the time series* The time series of intracity travel intensity can be decomposed into the trend, seasonal, and random components,

1 $$ Y_{t} = T_{t} + S_{t} + R_{t} , $$

where $Y_{t}$ is the observed time series data,${ }T_{t}$ is the trend component, $S_{t}$ is the seasonal component, $R_{t}$ is the random component, and subscript $t$ is the year 2019.

*Step 2 Prediction of the trend component* Assuming that the respective annual trends in 2019 and 2020 are similar (this assumption is supported by a Wilcoxon test with data in the duration without emergency response in 2019 and 2020), the trend component of 2020 can be seen as the trend component of 2019 plus an adjustment term representing the annual trend difference between 2019 and 2020. Before the emergency response in 2020, the trend component was normal and comparable with that of 2019. We took the average difference of duration in this period to represent the adjustment term using the formula:

2 $$ T_{t + 1} = T_{t} + \left[ {\mathop \sum \limits_{i = 1}^{n} \left( {T_{t + 1}^{^{\prime}} - T_{t}^{^{\prime}} } \right)} \right]/n, $$

where $t + 1$ is the year of Covid-19 and the emergency response policy; subscript $t$ is the year before the lockdown; $T_{t + 1}^{^{\prime}}$ is the trend component of travel intensity before the day of emergency response; $T_{t}^{^{\prime}}$ is the travel intensity trend of the same period of the lunar calendar in the previous year; $i$ is the day of that period; and $n$ is the length of that period.

*Step 3 Prediction of the seasonal component* As noted earlier, intracity travel intensity has a seasonality of seven days. We compared the decomposed seasonal component of 2019 and 2020 before the day of the emergency response using the Wilcoxon test and found a small difference. Therefore, we assumed that the seasonal component was the same before and after the emergency response, meeting the requirement of additive decomposition modeling. Hence, the seasonal component of 2020 was predicted using the seasonal component of 2020 before emergency response:

3 $$ S_{t + 1} = S_{t + 1}^{^{\prime}} , $$

where $S_{t + 1}$ and $S_{t + 1}^{^{\prime}}$ are the seasonal components after and before the date of the emergency response in $t + 1$ year, respectively.

*Step 4 Prediction of the random component* Similar to Step 2, we took the random component of 2020 after the emergency response as that of 2019 plus an adjustment term, which is determined by the average difference before the emergency response during the same period in 2019 and 2020:

4 $$ R_{{predict\left( {t + 1} \right)}} = R_{t} + \left[ {\mathop \sum \limits_{i = 1}^{n} \left( {R_{t + 1}^{^{\prime}} - R_{t}^{^{\prime}} } \right)} \right]/n, $$

where $R_{t + 1}^{^{\prime}}$ is the random component of travel intensity before the day of the emergency response and $R_{t}^{^{\prime}}$ is the random component in the same day of the week in the previous year.

*Step 5 Intracity travel intensity without emergency response* We formed the intracity travel intensity without emergency response by combining the forecast trend and the seasonal and random components:

5 $$ Y_{{predict\left( {t + 1} \right)}} = T_{{predict\left( {t + 1} \right)}} + S_{{predict\left( {t + 1} \right)}} + R_{{predict\left( {t + 1} \right)}} . $$

#### Estimation of Loss Rate of Intracity Travel Intensity

Intracity travel intensity is an index to reflect the city’s economic activities. The index of loss rate is calculated to further represent the impact of emergency response on the economy. After the prediction of intracity travel intensity without the emergency response, we estimated the loss rate of the intracity travel intensity by using the actual urban travel data observed during the emergency response as:

6 $$ {\text{TILR}} = {{\left[ {\sum {\left( {Y_{{predict\left( {t + 1} \right)}}  - Y_{{t + 1}} } \right)} } \right]} \mathord{\left/ {\vphantom {{\left[ {\sum {\left( {Y_{{predict\left( {t + 1} \right)}}  - Y_{{t + 1}} } \right)} } \right]} {\sum {Y_{{predict\left( {t + 1} \right)}} } }}} \right. \kern-\nulldelimiterspace} {\sum {Y_{{predict\left( {t + 1} \right)}} } }}.  $$

The above equation helps us calculate the loss rate of the intracity travel intensity of each city. We further summarize the loss rate at the provincial level by taking city scale GDP data as the weight ($PTILR$) in the equation:

7 $$ {\text{PTILR}} = \sum\limits_{{j = 1}}^{n} {\left[ {{{GDP_{j} } \mathord{\left/ {\vphantom {{GDP_{j} } {\left( {\sum\limits_{{j = 1}}^{n} G DP_{j} } \right)}}} \right. \kern-\nulldelimiterspace} {\left( {\sum\limits_{{j = 1}}^{n} G DP_{j} } \right)}} \times TILR_{j} } \right]} ,   $$

where subscript $j$ is the city within the province and superscript $n$ is the number of cities within the province.

## Results

We show the use of the prediction model developed in the previous section to predict the travel intensity without emergency response in 2020. We estimated the loss rate of intracity travel intensity of 360 cities and 33 regions in the first quarter of 2020, and analyzed the temporal and spatial distribution characteristics of the loss rate of intracity travel intensity.

### Loss Rates of Intracity Travel Intensity

**Fig. 2:**
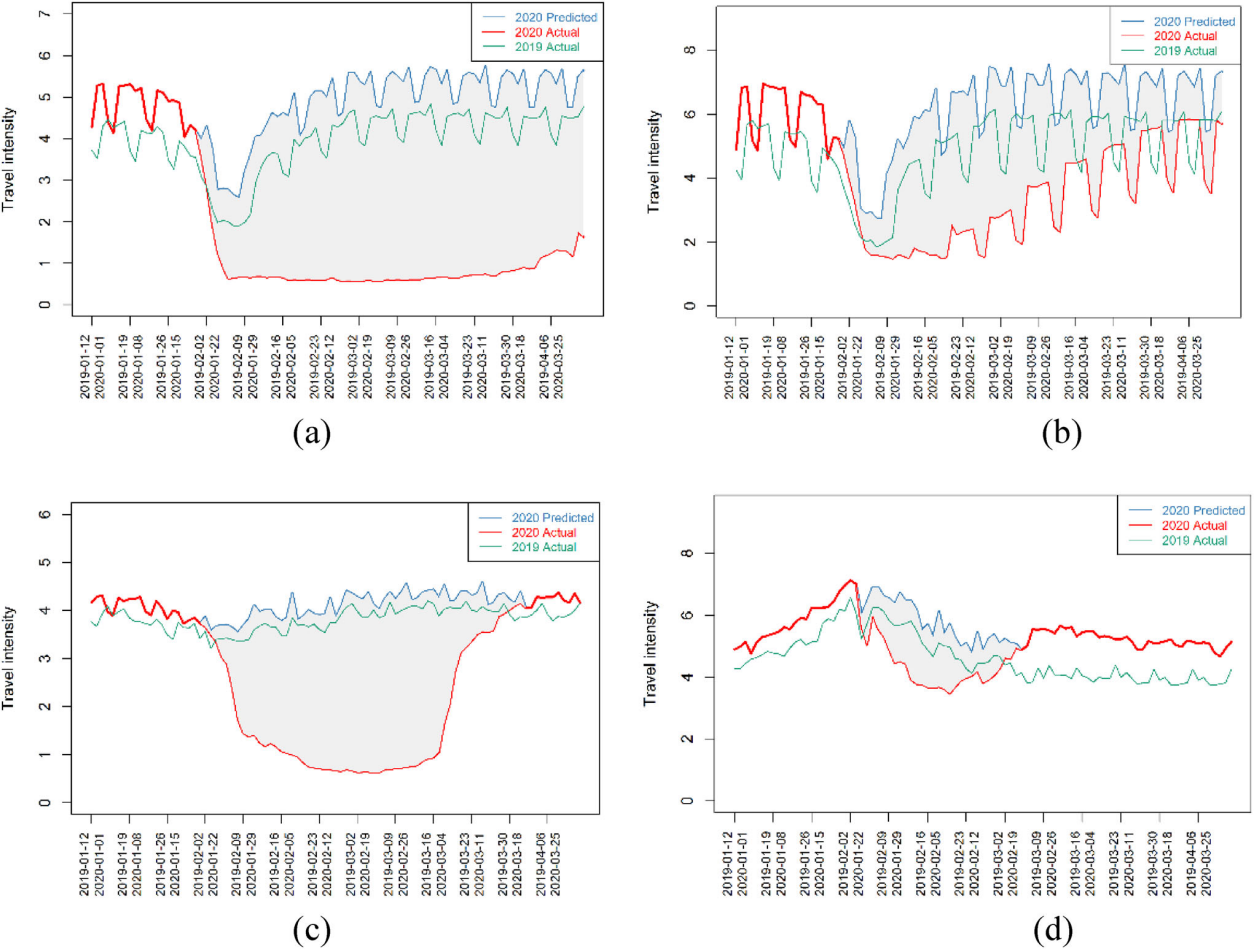
Typical observed and predicted time series of intracity travel intensity for **a** Wuhan, **b** Shanghai, **c** Bayingol, and **d** Zhaotong. *Note*: The data cover 1 January 2020 to 31 March 2020, and from 12 January 2019 to 12 April 2019. The time series in 2019 and 2020 are aligned according to the Chinese lunar calendar

The normal trend of intracity travel intensity is represented by the time series in 2019. Again, it has a seasonality of seven days. It dramatically decreased during the Spring Festival, but quickly bounced back six to seven days after the holiday. At the beginning of 2020, the trend of intracity travel intensity seems similar to the 2019 trend in the beginning of the year, but after the emergency response policy to Covid-19, intracity travel intensity in most cities fell rapidly. One or two days after the emergency response, the intracity travel intensity of each city dropped to its lowest point in history. Figure 2 shows the typical time series for Wuhan, Shanghai, Bayingol of Xinjiang, and Zhaotong of Yunnan Province in 2019 and 2020. The predicted trend in 2020 is also presented.

To clearly present the intracity travel intensity during the Spring Festival, the time series in 2019 and 2020 were aligned according to the Chinese lunar calendar. We found that the recovery speed is different for each city. At the end of March, Wuhan was just starting to recover, while Shanghai had almost recovered to the same level as the previous year. Bayingol showed stagnation, but recovered quickly at the end of March, and Zhaotong was only slightly affected and recovered mid-February. Interestingly, while Wuhan implemented the strictest policy of emergency response, Shanghai opted for a softer policy censuring unnecessary travel. Bayingol and Zhaotong had different levels of emergency response, a trend we observed for small cities. The gray area between the predicted and actual trends is the loss owing to Covid-19. Based on this definition, we calculated the loss rate in the first quarter of 2020 for the 360 cities. For the four cities mentioned above, we found that the loss rate of intracity travel intensity in Wuhan in the first quarter of 2020 is as high as 64.35%, followed by 34.72% for Shanghai, 37.36% for Bayingol, and 8.62% for Zhaotong.

### Spatial Distribution of Intracity Travel Intensity Loss Rate

**Fig. 3:**
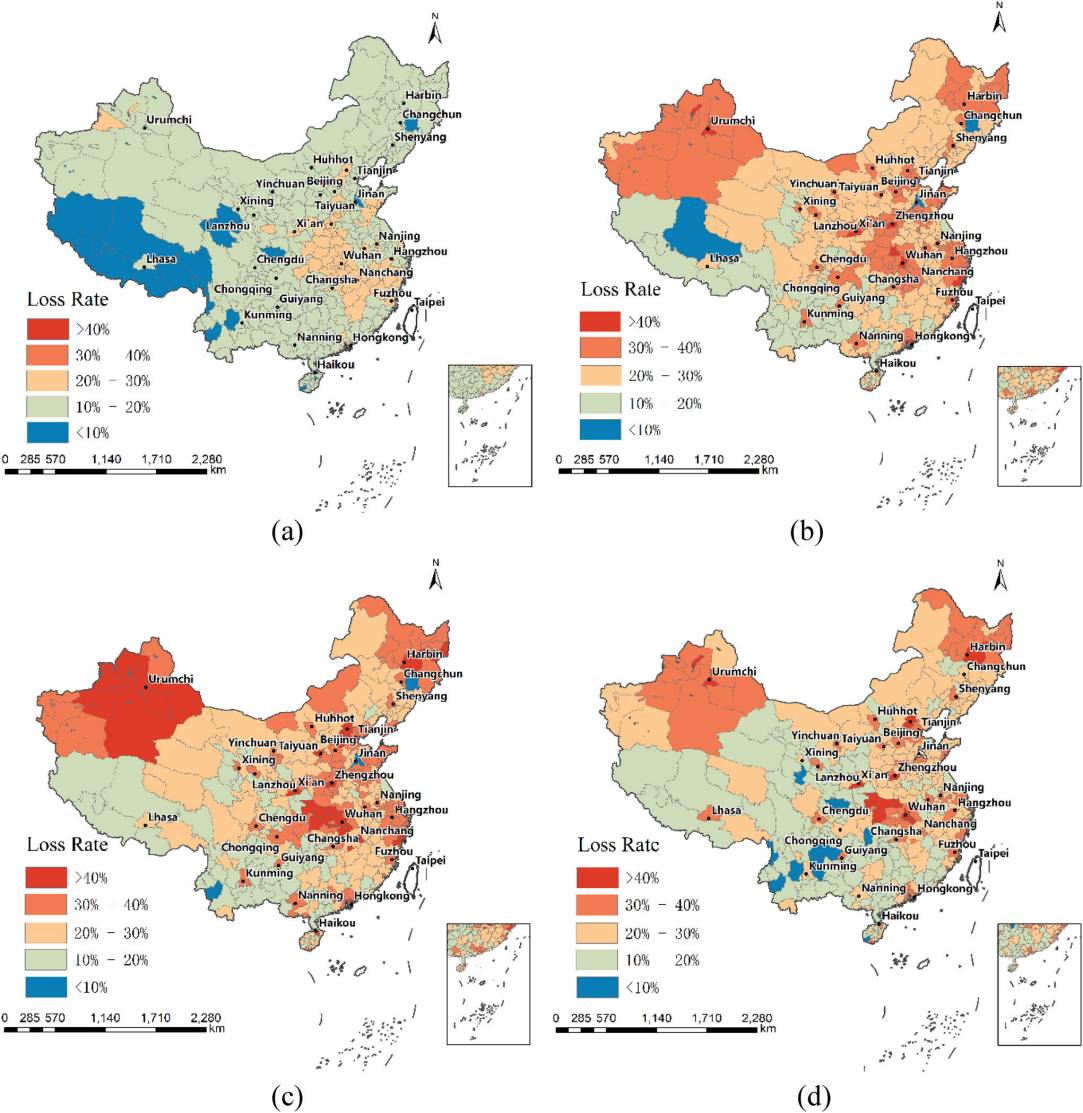
Spatial distribution of the loss rate of the intracity travel intensity in 360 cities. **a** 15 days after emergency response; **b** 30 days after emergency response; **c** 45 days after emergency response; **d** end of the first quarter of 2020

The result of the loss rate spatial distribution of the intracity travel intensity is shown in Fig. 3. The provincial capital city was the most affected area in a province. Indeed, 85% of the loss rates of provincial capital cities were larger than 30%. Within a province, the loss rates descend from the capital city to small cities. The loss rates for the top five cities in the first quarter of 2020 are as follows: 64.35% for Wuhan, 43.66% for Beijing, 43.62% for Urumchi, 43.00% for Harbin, and 41.25% for Zhengzhou. All these cities are located in northern or central China; those in the south and southwest were hardly affected.

**Fig. 4:**
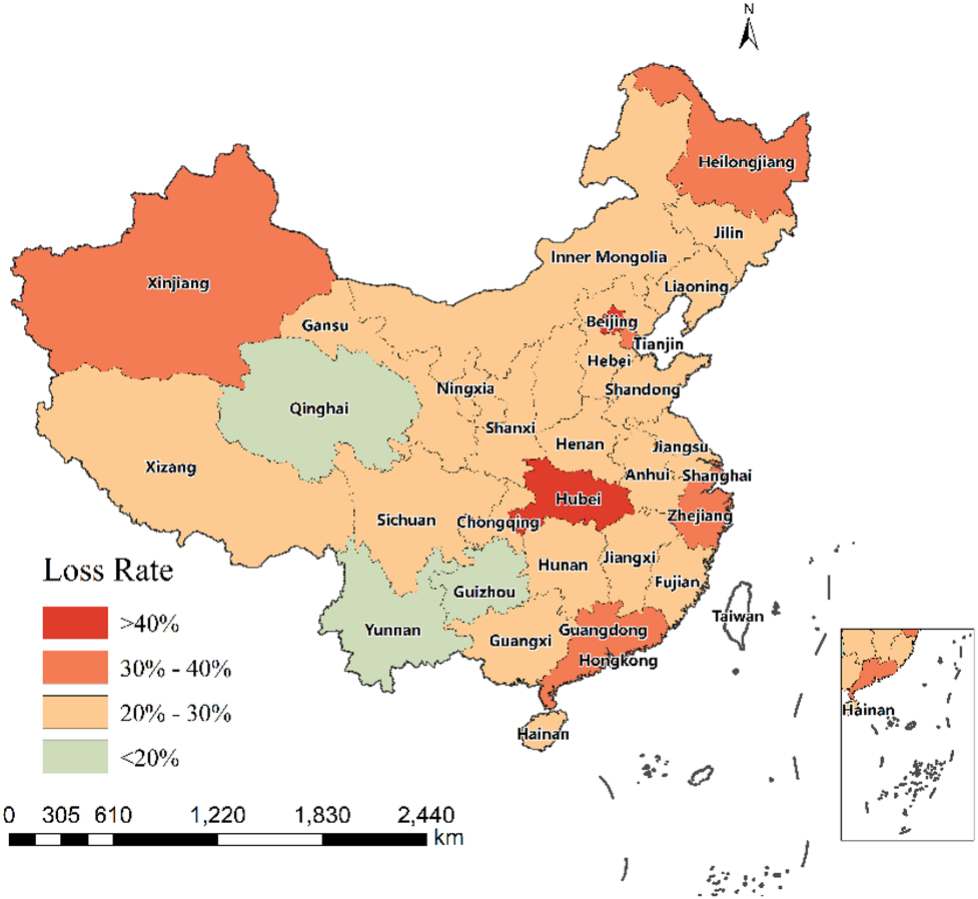
Spatial distribution of the loss rate of the intracity travel intensity in 33 provincial-level regions in the first quarter of 2020

Taking the city scale GDP of each city in 2019 as the weight and following Eq. 7 in Sect. 2.2.3, we aggregated the loss rate of the intracity travel intensity of 33 provinces (Fig. 4 and Table 1). At the provincial level, the loss rate of 33 regions ranges from 16.04 to 47.12%. We found great regional variation in the loss rate as well. For example, Hubei Province, which was the most severely affected by Covid-19, has the highest loss rate of 47.12%. Beijing, Heilongjiang, Xinjiang, and Tianjin followed, with a loss rate exceeding 37%. Qinghai, Guizhou, and Yunnan were the least affected, with less than 20%.

**Table 1:** Assessment results of the loss rates of intracity travel intensity for 33 provincial-level regions in the first quarter of 2020

Regions	Hubei	Beijing	Heilongjiang	Xinjiang	Tianjin
Loss rate	47.12%	43.66%	37.82%	37.55%	37.32%
Regions	Shanghai	Macao	Guangdong	Zhejiang	Hebei
Loss rate	34.72%	34.61%	30.78%	30.66%	29.77%
Regions	Henan	Chongqing	Ningxia	Jiangsu	Shaanxi
Loss rate	29.54%	28.57%	28.07%	27.65%	27.58%
Regions	Jiangxi	Hong Kong	Shanxi	Inner Mongolia	Fujian
Loss rate	26.80%	26.61%	26.34%	26.26%	26.22%
Regions	Liaoning	Hainan	Anhui	Jilin	Shandong
Loss rate	25.93%	25.49%	23.55%	23.39%	23.37%
Regions	Sichuan	Gansu	Guangxi	Hunan	Tibet
Loss rate	21.92%	21.29%	21.27%	21.25%	20.72%
Regions	Qinghai	Guizhou	Yunnan		
Loss rate	19.38%	17.93%	16.04%		

### Temporal Trend of Intracity Travel Intensity Loss Rates

**Fig. 5:**
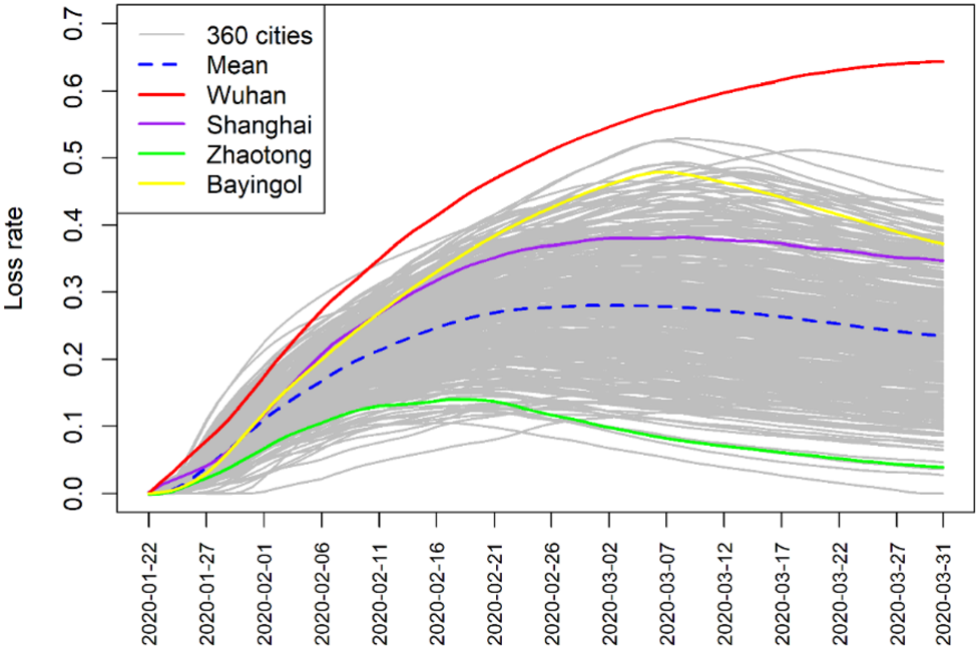
Temporal variation of the loss rate of the intracity travel intensity in 360 cities of China

Figure 5 shows the time series of the loss rates for the 360 cities. The fundamental trend is that the loss rates increase quickly after the emergency response, then gradually stabilize, and finally decrease. Wuhan shows the largest loss rate with no decreasing trend in the first quarter. The average loss rate at the end of the first quarter is around 0.2, which reflects a 20% fall in intracity travel intensity in China in the first quarter of 2020, owing to reduced social and economic activities. The loss rate curves of Shanghai, Zhaotong, and Bayingol are illustrated in Fig. 5 to show the regional heterogeneity.

**Fig. 6:**
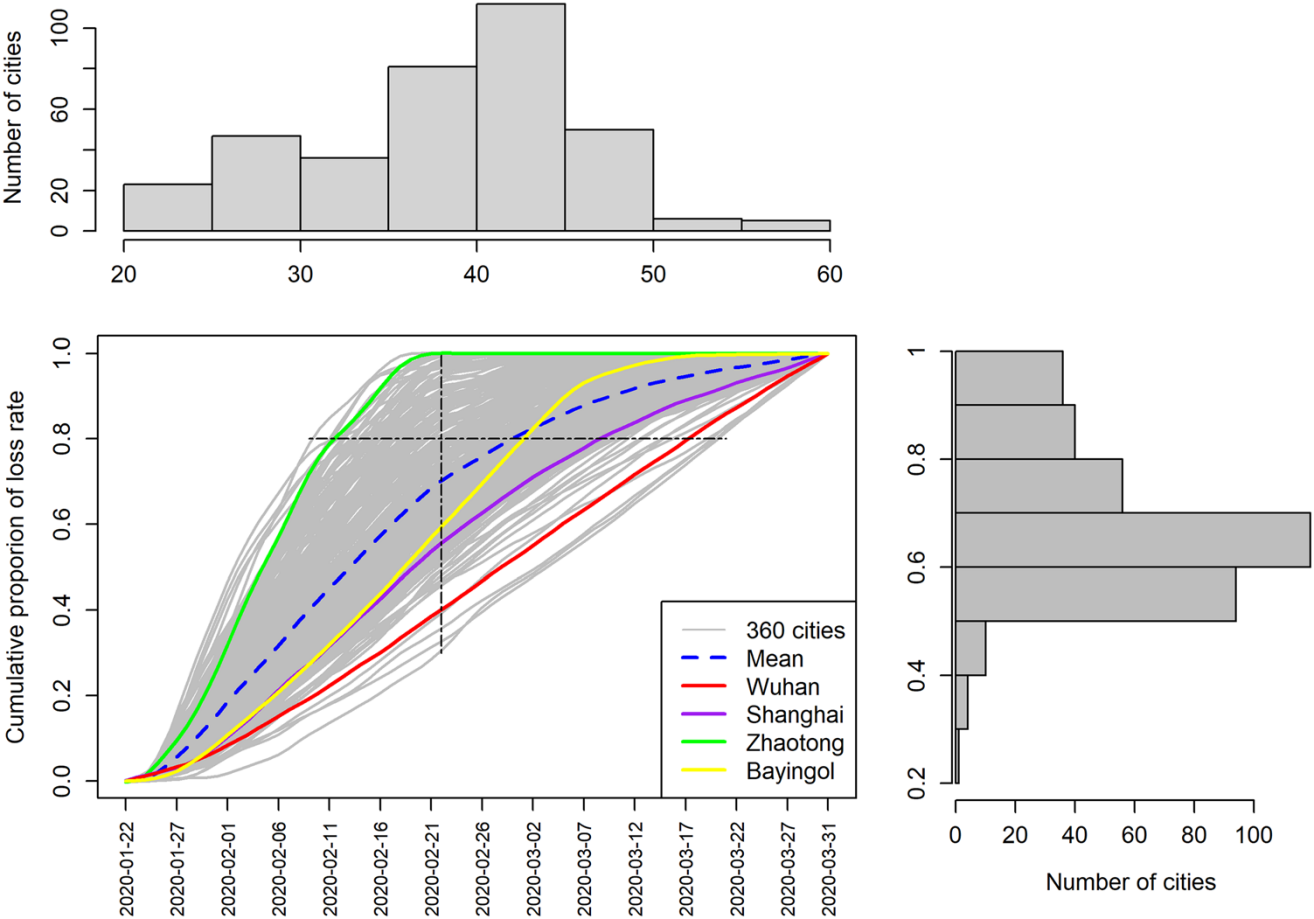
Temporal trend of cumulative proportion of the loss rate in 360 cities of China. The horizontal dashed line fixed the cumulative proportion of loss rate at 0.8, which generates the upper histogram profile, and the x-axis of the upper histogram is time (days); The vertical dashed line fixed the time at 30 days after the emergency response, which generates the right histogram profile, and the y-axis of the right histogram is cumulative proportion of loss rate

Figure 5 shows an assessment of the absolute loss rate. We recalculated the loss rate trend to represent its cumulative proportion with time increasing in order to more clearly reflect the situation of a city (Fig. 6). The range of cumulative proportion of the loss rate is between 0 and 1 (100%). It is a standardized value (make all loss rate range from 0 to 1). When the cumulative loss rate reaches 1, it proves that the city has ended incurring losses and recovered to normal state at this moment.

Zhaotong (green line in Fig. 6), a city in Yunnan Province, reached a value of 1 (100%) within one month, meaning that this city recovered to the normal state in a short time. In contrast, Wuhan (red line in Fig. 6) reached 1 (100%) only by the end of the first quarter of 2020, meaning that it was not recovered to the normal state in the first quarter. The average trend of the cumulative proportion of loss rate (blue line in Fig. 6) provides an overview of recovery in all of China. The upper histogram shows the distribution of the times when the cities’ cumulative proportion of loss rate reached 0.8. It means that 40 days after the emergency response, half of the 360 cities reached 80% of their total loss, and 45 days after the city lockdown, 83% of the cities reached 80% of their total loss. The right histogram in Fig. 6 shows the change from another perspective. When fixing the time at 30 days after the emergency response, we found that 70% of the cities reached a loss of more than 60%, 21% of the cities reached a loss of more than 80%, and 10% of the cities reached a loss of more than 90%. According to the definition of loss in Fig. 1, when a city reached all of its loss, it means that the city has recovered to the normal state. Therefore, we can interpret the cumulative proportion of loss rate as the rate of recovery to normal state—from the perspective of recovery, 30 days after the emergency response, 21% of the cities saw over 80% recovery and 10% of the cities showed more than 90% recovery; 45 days after the emergency response, more than 83% of the 360 cities witnessed 80% recovery.

**Fig. 7:**
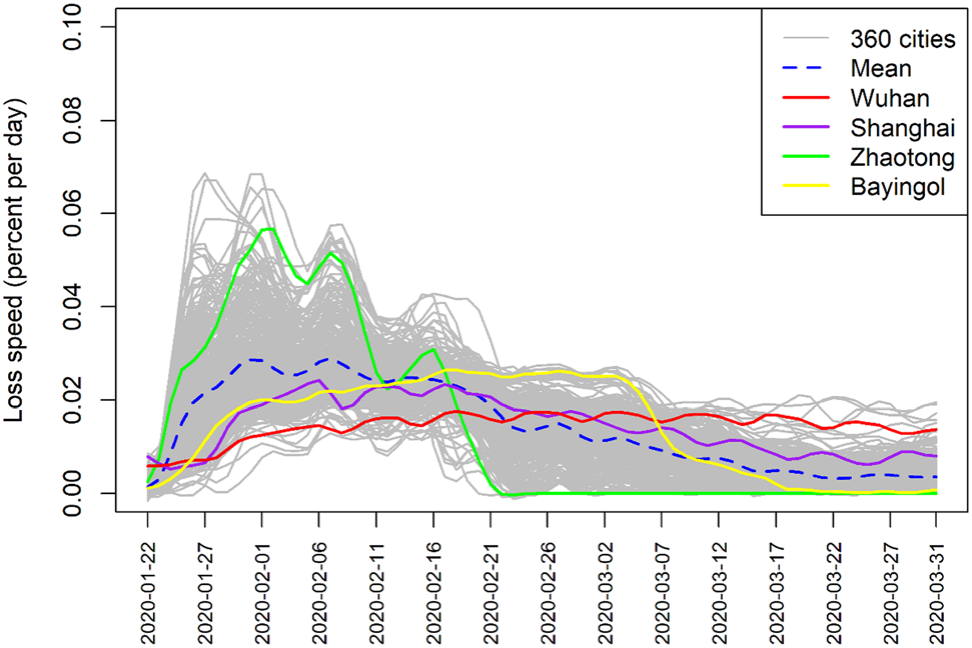
Loss speed chart of intracity travel intensity in 360 cities of China

The first-order derivative of the cumulative proportion curve represents the speed of loss (Fig. 7). We identify some patterns of loss here as well. Zhaotong is represented by a green curve indicating a fast loss and fast recovery pattern. Wuhan’s loss (red curve) shows no recovery pattern. Wuhan maintained a stable loss speed until the end of first quarter, while Shanghai’s loss shows a gradual recovery pattern, like most cities in China; this is the average curve trend. Bayingol fared similar to Zhaotong, although its loss speed remained high for several days, reaching its loss before the end of first quarter, and completing its recovery.

## Discussion

We mainly discuss whether or to what extent the loss rate of intracity travel intensity reflects the economic impact of emergency response, and the spatial and temporal patterns of the effect of the emergency response.

### Validation of Intracity Travel Intensity Loss Rate

**Fig. 8:**
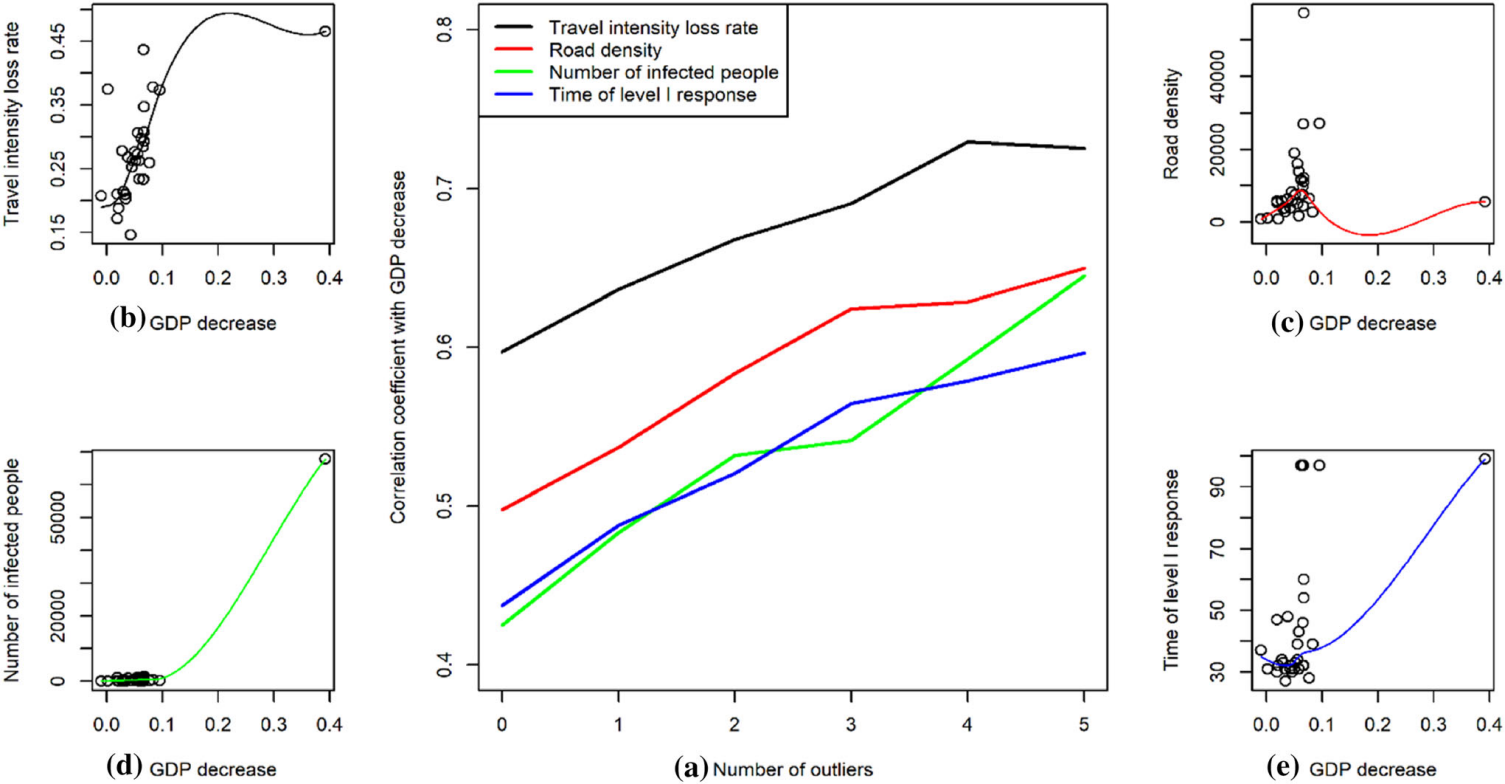
Correlation analysis results of gross domestic production (GDP) decrease rate and various indicators (Pearson’s linear correlation using R version 4.0.3). **a** Allows removing outliers from the correlation analysis to capture the trend of correlation. The number of outliers means how many samples were excluded from correlation analysis. The other four subfigures are scatter plots that depict the relationship between the GDP decrease rate and **b** loss rate of intracity travel intensity, **c** road density, **d** number of Covid-19 infection cases, and **e** duration of public health emergency response

We developed the intracity travel intensity loss rate to assess the economic effect of the emergency response. There are other data available to measure this effect, such as the number of Covid-19 infection cases (Grasselli et al. 2020; Zhang, Xiao, et al. 2020) and the duration of the public health emergency response (Shrivastava et al. 2015; Craig et al. 2018); road travel and density can also be used as an indirect index (Cowie et al. 2016; Yang, Yin, et al. 2016a). These data are easier to obtain, allowing us to demonstrate the superiority of the intracity travel intensity loss rate. We thus conducted a correlation and comparison analysis. We took the officially published provincial-level GDP growth rate of 33 regions in the first quarter of 2020 as a direct measure of the economic effect of Covid-19; the correlations of provincial-level GDP decrease with the loss rate of the intracity travel intensity, number of Covid-19 infection cases, duration of public health emergency response, and road density are presented in Fig. 8. We considered the potential impact of outliers that have a large difference between common values, and adopted a dynamic correlation analysis strategy to capture the general trend of correlation. If no data are treated as outlier, the correlation between intracity travel intensity loss rates and GDP is around 0.6; the others are all less than 0.5. If there are data treated as outliers, the aforementioned correlation may strengthen. The scatter plots also show a clear linear relationship, unlike the other relationships.

### Relationship Between Intracity Travel Intensity Loss Rate and Gross Domestic Production Loss Rate

**Fig. 9:**
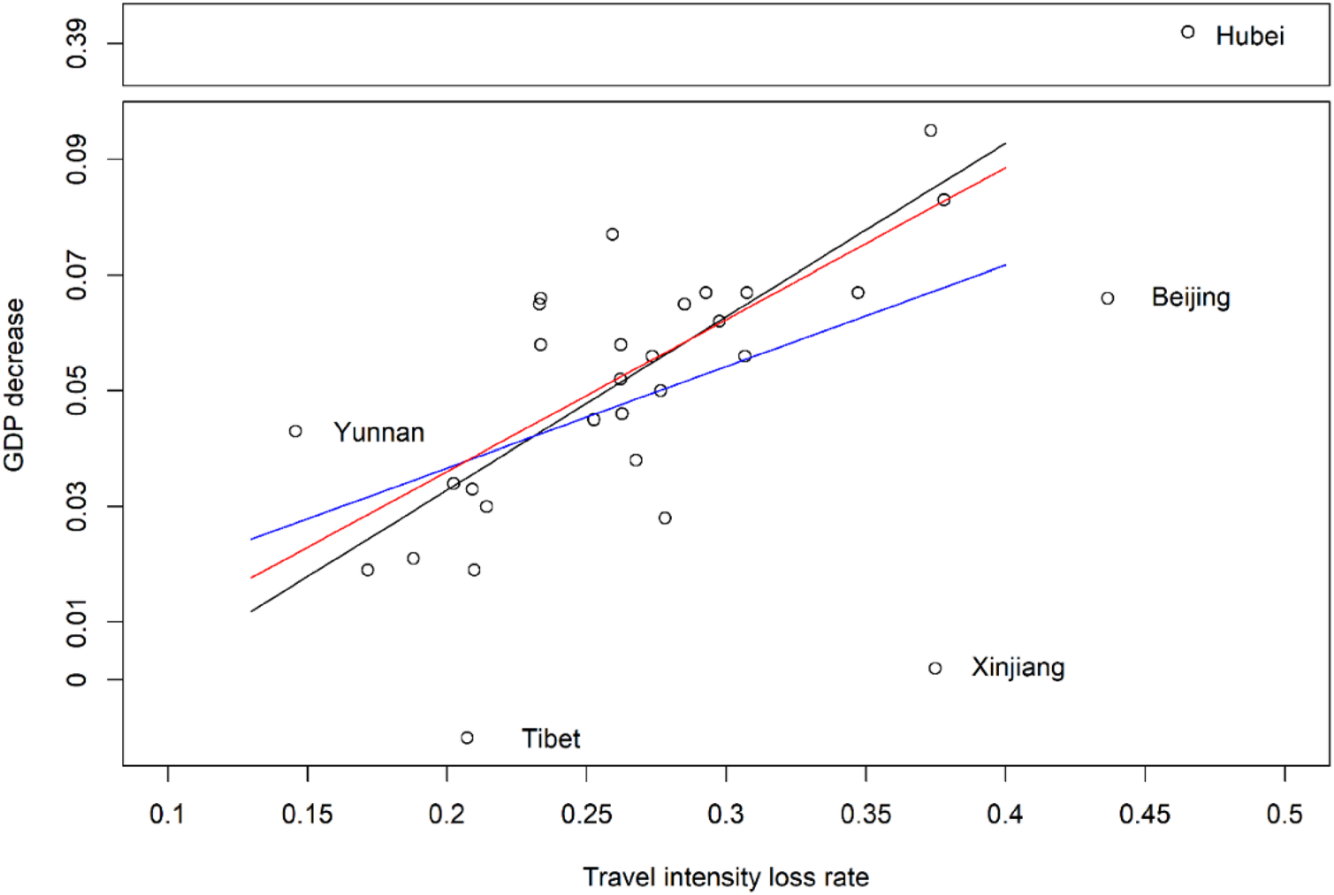
Scatter plot between the rate of decrease in gross domestic production (GDP) and the rate of loss in travel intensity. *Note*: Blue line—Regression 1, removing Hubei for special discussion; Red line—regression 2, removing Hubei, Beijing, Xinjiang, and Tibet for special discussion; Black line—regression 3, removing Hubei, Beijing, Xinjiang, Tibet, and Yunnan for special discussion

We proved that the loss rate of the intracity travel intensity could be a possible approximate index that represents the GDP loss rate. We now discuss the degree to which it can reflect the economic effect and whether it is significant. According to the principle of parsimony in statistical modeling, a sample linear regression is conducted. Figure 9 and Table 2 illustrate the results of a simple regression.

**Table 2:** Results of regression between travel intensity loss rate and gross domestic production (GDP) decrease rate

	Regression 1		Regression 2		Regression 3	
	*n* = 32 **(**Adjusted *R*^2^ = 0.212)		*n* = 29 **(**Adjusted *R*^2^ = 0.539)		*n* = 28 (Adjusted *R*^2^ = 0.593)	
	Intercept	Travel intensity loss rate	Intercept	Travel intensity loss rate	Intercept	Travel intensity loss rate
Coefficients	0.002	0.175	− 0.016	0.263	− 0.027	0.300
Std. Error	0.016	0.059	0.012	0.047	0.013	0.049
t-statistic	0.096	2.969	− 1.322	5.603	− 2.052	6.115
p-value	0.924	0.006	0.198	7.8e−06	0.051	2.5e−06
[95% CI]	[− 0.032, 0.035]	[0.054, 0.297]	[− 0.042, 0.009]	[0.166, 0.359]	[-0.054, 0]	[0.199, 0.401]

Regression 1—Removing Hubei for special discussion; Regression 2—Removing Hubei, Beijing, Xinjiang, and Tibet for special discussion; Regression 3—Removing Hubei, Beijing, Xinjiang, Tibet, and Yunnan for special discussion

Hubei is an outlier of the sample data because the ratio between its official GDP decrease and intracity travel intensity loss rate varies too much from those of other provinces. Taking out this outlier, we plotted the regression result using a blue line. Both the F-test for linearity and t-test for parameters are significant, but the adjusted R^2^ is only 0.21. Thus, only 21% of the variance of the variable GDP decrease can be explained by the loss rate of the intracity travel intensity.

To refine the model, we considered more outliers. If we remove Hubei, Beijing, Xinjiang, and Tibet, the R^2^ can reach 0.54, as shown by the red line. If we further remove Yunnan, the R^2^ can reach 0.6, meaning that 60% of the variance of the variable GDP decrease can be explained by the loss rate of the intracity travel intensity, as shown by the black line. The regression reflects a common relationship between the GDP decrease and the loss rate of the intracity travel intensity in China. Removing some province-level data points means that the relationships in these provinces need to be specifically discussed. When using the intracity travel intensity loss rate to represent the GDP decrease, both common trend and special cases need to be discussed.

Hubei was the most affected by Covid-19. The number of infection cases reached 67,800 at the end of March (Maier and Brockmann 2020), forcing the province to impose a stringent emergency response that was not eased until 2 May 2020. Its capital city Wuhan was under lockdown from 23 January to the end of March. Nearly all substantial economic activities stopped. Other cities in Hubei Province also faced a similar situation. Therefore, the loss rates of both real GDP and our calculated intracity travel intensity are very high.

Compared with other provinces, the ratio between the GDP loss rate and the loss rate of the intracity travel intensity is lower for Beijing, Xinjiang, and Tibet. This means that the GDP loss rates were smaller than our expectation under the situation of current high intracity travel intensity loss rate. One possible explanation is that their economies are not as dependent on city traffic systems as are those of other provinces. The main industries in Beijing, the capital of China, are finance, information, science, and education, which are not so sensitive to the city traffic system (Wang et al. 2017). In Xinjiang and Tibet, however, the density of population is lower and, thus, Covid-19 had a relatively lower effect on public confidence (Muniz-Rodriguez et al. 2020). Their secondary industries, including raw material processing and mining, increased during this time, which eased GDP losses. In contrast, Yunnan was hit harder than we expected, probably because more than half of its GDP comes from tourism and related industries, which are sensitive to the city traffic system. Except for these provinces, we can still propose a common relationship between the loss rates of the GDP and intracity travel intensity: That is, every one unit increase of the loss rate of intracity travel intensity will cause a 0.300 [0.199, 0.401] unit increase in the GDP loss rate at the 95% significance level.

Interestingly, for all provinces, the GDP loss rates are higher than our estimated loss rates of the intracity travel intensity. This result may be because the online economy also plays an important role in China’s overall economy. We argue that the online economy is more independent of intracity travel than traditional industry. Then, the decrease in travel activities led to the decline in GDP, but the development of the online economy during the pandemic made up for the decline in GDP to some extent. In this study, we can only measure the decline in GDP caused by reduced travel and cannot measure the economic contribution of the online economy. Therefore, future research needs to explore further the economic contribution of the online economy during Covid-19.

### Spatial and Temporal Effect of the Emergency Response

The results motivate a discussion about the spatial and temporal patterns of the effect of the emergency response. First, in a province, the spatial effect radiated in intensity from the center, the capital city, and moved outward. This is because the capital city is often also the economy and political center of a province. At the provincial level, we do not see a clear radiating pattern, because the countermeasures are not the same among provinces. The spatial effect of emergency response seems to be consistent with the findings of previous studies on the spatial distribution of Covid-19 outbreaks. For instance, Ning et al. (2021, p. 1) argued that the Covid-19 epidemic showed a spatial pattern of “multi-center agglomeration distribution” in China. Liu, Fang, et al. (2020) and Xie et al. (2020) found that outbreak-related emergencies often have a greater impact on the economically developed provincial capitals.

Second, the temporal trend of the intracity travel intensity loss rate reflects a full loss and recovery pattern. Indeed, the general pattern is a sudden loss, with a race to the bottom and then gradual recovery. Most cities, especially provincial capital cities, followed this pattern. The pattern further changes for some small cities, where the trend is a quick loss and a quick recovery, as in Zhaotong and Bayingol (quick loss lasting over a long period followed by a sudden recovery). These patterns reflect the effect of the emergency response and the resilience of cities.

## Conclusion

Intracity travel intensity data are a new type of big data. They help researchers solve conventional problems such as loss estimation, our primary focus here. We validated our proposed method using GDP data and confirmed its feasibility for estimating rapid loss during disasters. Compared with other indices, such as the number of Covid-19 infection cases or the duration of the emergence response, we argue that the loss rate calculated from intracity travel intensity data can better explain changes in GDP growth data. Indeed, it can effectively reflect the full pattern of a city’s loss and recovery. Although it is an approximate estimation of economic loss, the method can yield a fast, real-time estimation of loss to support decision making.

The economic effects of Covid-19 during its early stages on Hubei, Beijing, Xinjiang, Tibet, and Yunnan should be discussed specifically because the relationship between GDP and intracity travel intensity in these provinces is different from what was seen in other provinces. Except for these provinces, a common relationship between the loss rates of GDP and intracity travel intensity is proposed.

Cities can recover faster even under the effect of an emergency response, especially when both citizenry and the government work toward reaching a normal state. We confirmed that the temporal trend shows a pattern of loss and recovery in China. While the general pattern among most cities (especially provincial capitals) is one of a sudden loss, a drop to the bottom, and then gradual recovery, small cities show more heterogeneity in trends.

Nevertheless, the index of the loss rate of the intracity travel intensity can only reflect the economic damage caused by traffic limitations, which makes the difference between it and the real GDP loss rate worthy of further study. This difference can reflect the effectiveness of other recovery policies or countermeasures during an emergency response—for example, group buying and online business. We, therefore, recommend these topics for further investigation.

## Footnotes

1 https://qianxi.baidu.com/.

2 https://www.webmap.cn/.
